# Sex Differences and Adverse Effects between Chemotherapy and Immunotherapy for Non-Small Cell Lung Cancer

**DOI:** 10.7150/jca.40196

**Published:** 2020-03-05

**Authors:** Theodora Tsiouda, Chrisanthi Sardeli, Konstantinos Porpodis, Maria Pilikidou, Georgios Apostolidis, Krystallia Kyrka, Angeliki Miziou, Konstantina Kyrka, Zoi Tsingerlioti, Souzana Papadopoulou, Anta Heva, Charilaos Koulouris, Dimitrios Giannakidis, Konstantina Boniou, Isaak Kesisoglou, Anastasios Vagionas, Christoforos Kosmidis, Christina Sevva, George Papazisis, Alexandru Marian Goganau, Konstantinos Sapalidis, Kosmas Tsakiridis, Stavros Tryfon, Michalis Platanas, Sofia Baka, Bojan Zaric, Branislav Perin, Savvas Petanidis, Paul Zarogoulidis

**Affiliations:** 1 Pulmonary Department, “Theageneio” Cancer Hospital, Thessaloniki, Greece.; 2 Department of Pharmacology & Clinical Pharmacology, School of Medicine, Faculty of Health Sciences, Aristotle University of Thessaloniki, Thessaloniki, Greece.; 3 Pulmonary Department, G. “Papanikolaou” General Hospital, Aristotle University of Thessaloniki, Thessaloniki, Greece.; 4 3rd Department of Surgery, “AHEPA” University Hospital, Aristotle University of Thessaloniki, Medical School, Thessaloniki, Greece.; 5 General Surgery Clinic 1, University of Medicine and Pharmacy of Craiova, Craiova County Emergency Hospital, Craiova, Romania.; 6 Thoracic Surgery Department, “Interbalkan” European Medical Center, Thessaloniki, Greece.; 7 Pulmonary Department (NHS), G.H. “G. Papanikolaou” Thessaloniki, Thessaloniki, Greece.; 8 Urology Department (NHS), General Hospital of Giannitsa, Giannitsa, Greece.; 9 Oncology Department, “Interbalkan” European Medical Center, Thessaloniki, Greece.; 10 Institute for Pulmonary Diseases of Vojvodina, Faculty of Medicine, University of Novi Sad, Serbia.; 11 Department of Pulmonology, I.M. Sechenov First Moscow State Medical University, Moscow, Russian Federation.

**Keywords:** Lung cancer, chemotherapy, immunotherapy, adverse effects

## Abstract

**Introduction**: Lung cancer remains the leading cause of cancer mortality in men and women and around the world. Approximately 90% of cases of lung cancer are caused by smoking and the use of tobacco products. However, other factors such as asbestos, air pollution and chronic infections can contribute to pulmonary carcinogenesis. Lung cancer is divided into two broad histological categories, which develop and spread different small cell lung carcinomas and non-small cell lung carcinomas. The treatment options for lung cancer include surgery, radiotherapy, chemotherapy and targeted treatments. Tumor induced immune suppression is vital for malignant progression. Immunotherapies act by strengthening the patient's innate tendency for an immune response and give positive promise to patients with non-small cell lung cancer and small cell lung cancer. Immune checkpoint inhibitors are a new approach to cancer therapies. Just as immune therapies include a new approach to cancer biology, the toxicities associated with these factors have created new challenges in clinical practice.

**Materials & Methods**: Patients (218) aged 40-80 years were treated with either chemotherapy or immunotherapy. Their response to treatment and any subsequent adverse drug reactions were studied.

**Results**: 69% of patients were treated with chemotherapy and 31% were treated with immunotherapy. The type of treatment had a statistically significant effect on the undesirable effects of the treatment.

**Conclusions**: The type of treatment was statistically significant in responding to the treatment and treatment side effects but not in the rate of death.

## Introduction

Lung cancer is still diagnosed at a late stage due to lack of early disease symptoms. We have novel diagnostic equipment such as radial endobronchial ultrasound, convex probe endobronchial ultrasound, electromagnetic navigation and cone beam ct bronchoscopy 1-3. At advance stage disease we need tissue biopsies for non-small lung cancer patients in order to investigate the expression of a number of genes which are associated with the treatment options of a patient 4-6. In specific we have to investigate the expression of epidermal growth factor (EGFR), anaplastic lymphoma kinase (ALK), proto-oncogene B-Raf (BRAF), proto-oncogene tyrosine-protein kinase-1 (ROS1) and programmed death-ligand-1 (PD-L1). The EGFR, ALK, BRAF and ROS1 gene expression is associated with tyrosine kinase inhibitors (TKIs) 7,8. There is also the T790 mutation which is associated with a new generation TKI the osimertinib 9. The programmed death-ligand-1 (PD-L1) expression is associated with immunotherapy drugs 10. If a patient is not a candidate for targeted treatment with TKIs or immunotherapy then chemotherapy is still an option as first line treatment. Regarding small cell lung cancer (SCLC) curently platinum analogues and etoposide still remains the tip of the arrow as first line treatment 11. In the past year immunotherapy for sclc as first line treatment has been also introduced 12. All treatments have their advantages and disadvantages. Regarding chemotherapy we have observed fatigue, myalgia, athralgia, anemia, esophagitis and neutrapenia 13. The tyrosine kinase inhibitors have pneumonitis, esophagitis and skin rash which is associated in most cases with the dosage 14,15. Immunotherapy has orogonitis, pneumonitis, athritis, vitiligo, resurgence of hepatitis and disregulation of the thyroid gland 6,16 An aspect that has not been fully investigated is the differences of adverse effects, gender and disease response between chemotherapy and immunotherapy 17-21. In the current research paper we investigated the differences of adverse effects between chemotherapy and immunotherapy in first line treatment for squamous cell carcinoma. Those patients that had PD-L1 ≤50% received chemotherapy doublet.

## Patients and Methods

The study was approved by the Investigational Review Board (IRB) of the General Cancer Hospital “Theageneio”, Thessaloniki, Greece. Initially, reference is made to the general characteristics of the sex and age of the patients. The medical data of the sample, the treatment of chemotherapy or immunotherapy, the concomitant diseases, the treatment response, whether the patient has died and the complications of the treatment are presented. Based on the complications reported for the patients, a re-coding of the data in which the main adverse events were selected was performed to examine whether gender is a statistically significant effect factor. In addition, it was examined whether sex had a statistically significant effect on the treatment and death rate, and whether the type of treatment received (chemotherapy or immunotherapy) had an effect on treatment response, death rate and treatment complications. The mean body mass index (BMI) was 25. Inclusion criteria were newly diagnosed (first line) squamus cell nsclc patients with PD-L1 expression available. Patients with PD-L1 expression ≤50% received carboplatin and nab-paclitaxel and patients with PD-L1 ≥50% received pembrolizumab. All patients were ≥18-70 years old and were fit to receive the previously mentioned treatment options according to the drugs instructions 22.

**Figure 1:**
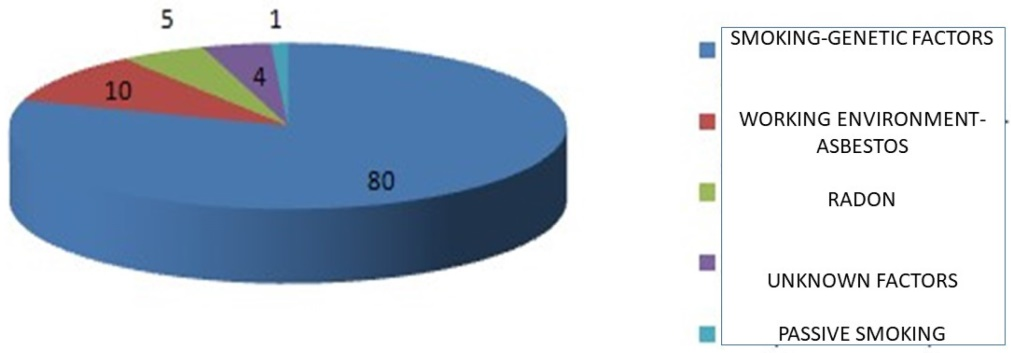
Key contributors to pulmonary carcinogenesis.

54.8% of the sample consisted of men (N = 119), and women constituted 45.2% (N = 217) (Figure 1).

**Figure 2:**
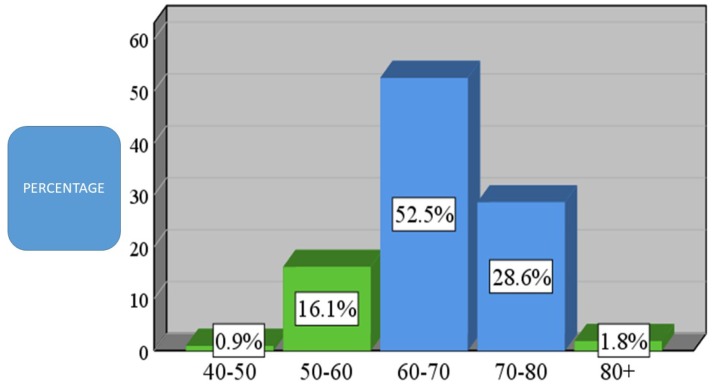
Age demographic of patients present in the current study (N = 336).

82.9% of the total sample was aged 60 years and older. Analytically, 52.5% were from 60 to 70 years old (N = 114), 28.6% were from 70 to 80 years old (N = 62), and 1.8% were over 80 years old. In addition, 16.1% were from 50 to 60 years of age and 0.9% were from 40 to 50 years old (Figure 2).

**Figure 3:**
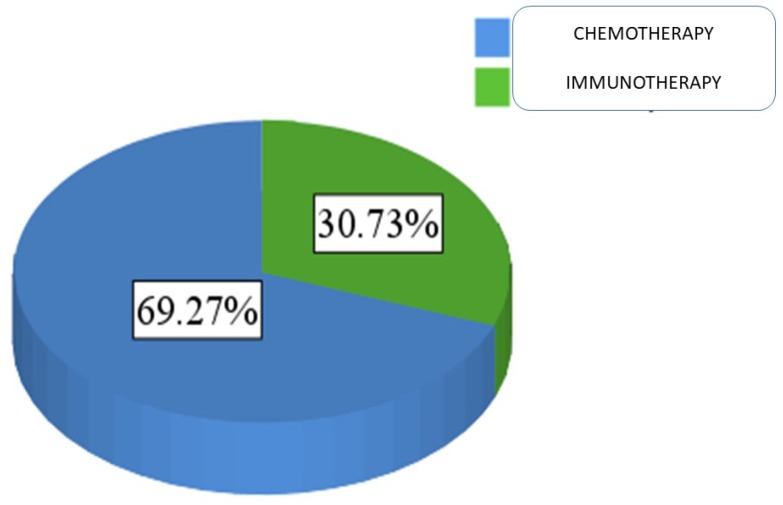
Proportion of patients administered either chemotherapy or immunotherapy.

For patients' medical data, 69.3% of the sample was treated with chemotherapy (N = 151), and 30.7% were treated with immunotherapy (N = 67) Figure 3.

The concomitant diseases reported for this patient sample are presented in order of priority in Table 1.

As shown in Table 1, chronic obstructive pulmonary disease was far from the first place of occurrence with 75.7%. Coronary artery disease (30.3%), arterial hypertension (29.4%), and diabetes mellitus (28.9%) were followed at the next post in the co-morbid hierarchy. Other co-morbidities also included (in order of rank hierarchy) were chronic illnesses (9.6%), chronic renal failure (9.2%), bronchial asthma (5%), hypothyroidism (5%) and stroke (3.2%). Remaining concomitant diseases were heart failure (2.3%), hyperlipidemia (2.3%), autoimmune diseases (1.4%), gastroesophageal reflux (1.4%) and hyperthyroidism (1.4 %).

**Figure 4:**
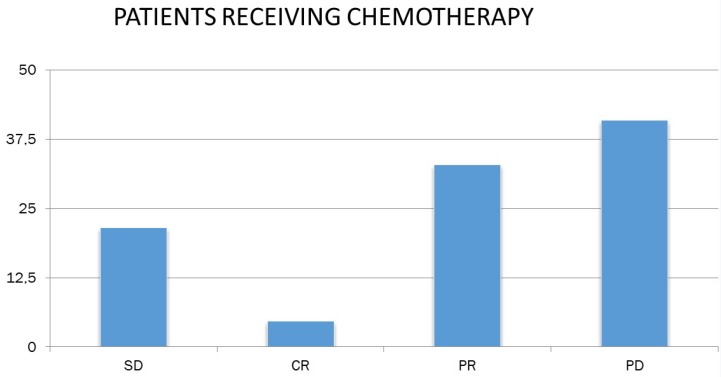
Patient response to chemotherapy. SD: stable disease; CR: complete remission; PR: partial remission and, PD: progressively worsening disease.

Concerning the response of patients to chemotherapy and immunotherapy, 18.1% had stable disease (N = 39), 9.8% had complete remission (N = 21), 36.7% had partial remission N = 79) and 35.3% (N = 76) had progressively worsening disease (Figure 4).

**Figure 5:**
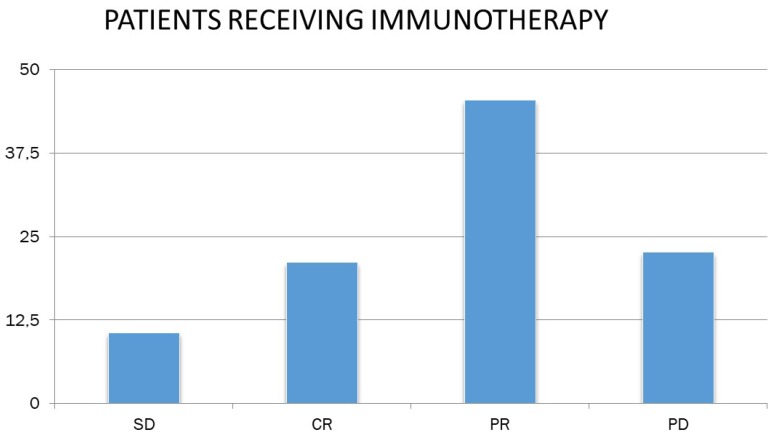
Patient response to immunotherapy. SD: stable disease; CR: complete remission; PR: partial remission and, PD: progressively worsening disease.

Studying the response to chemotherapy and immunotherapy separately, and given that patients in the chemotherapy-treated sample were more than doubled (N = 149) than patients receiving immunotherapy (N = 66), the following were found. 32.9% of patients receiving chemotherapy had partial remission (N = 49), and the corresponding rate for immunotherapy patients was 45.5% (N = 30). In addition, 21.5% of the chemotherapy patients (N = 32) and 10.6% of the receiving immunotherapy (N = 7) had stable disease. 21.2% of the receiving immunotherapy had complete remission (N = 14), whereas the corresponding rate for chemotherapy recipients was lower by 4.7 % (N = 7). Finally, 40.9% of the chemotherapy patients (N = 61) and 22.7% of the receiving immunotherapy (N = 15) had progressively worsening disease (Figure 5).

In total, 18.8% of the patients in this sample died (N = 41).

About the complications of treatment, N = 204 valid responses were given and 269 complications were reported. Table 2 lists all of the treatment complications reported. The most frequently reported complications were infections (14.2%), leg pain with 13.2%, pancytopenia with 12.7%, respiratory failure II with 9.8%, depression with 8.3 %, the incidence of diabetes mellitus II by 7.8%, hypothyroidism by 7.8%, gastrointestinal disorders by 6.9%, hyperthyroidism by 6.4%, anemia by 5.4%, cerebrovascular accident with 4.4% and neutropenia by 3.9%. These and other treatment complications reported are presented in Table 2.

In order to examine the main question of the study: "Is there a gender difference in the adverse effects of chemotherapy and immunotherapy in patients with lung cancer?”, data on the treatment complications were coded in a new variable, which was maintained only the most frequently reported complications of treatment. This procedure was performed to limit the number of treatment complications, with care that the size of each subgroup of treatment complications (N ≥ 5) is not too low so that the data is suitable for the use of the control statistic χ^2^ (chi square).

During the coding process, cases of co-morbidity were excluded in two of the most frequently occurring treatment complications, and only those cases where either the patient was reporting a single complication or the co-morbidity he was experiencing included a most frequently occurring complication and a second, lower frequency, complication. The valid sample for this control was N = 146, of which N = 78 were men and N = 68 were females. Gender was the independent variable and treatment complications were the dependent variable. Table 3 is a double-entry and presents the frequencies and percentages of the most frequently occurring treatment complications for each sex separately.

As shown in Table 3, leg ulcers were seen in 17.9% of men and 14.7% of women. Pancytopaenia and infections occurred in 17.9% of men and 10.3% of women, respiratory failure II in 11.5% of males and 8.8% of women, depression of 3.8% of men and 10.3% of women, and hypothyroidism was present in 11.5% of males and 7.4% of women.

Furthermore, the incidence of diabetes mellitus II was present in 10.3% of males and 2.9% of women, gastrointestinal disturbances did not occur at all in males and occurred in 16.2% of women, hyperthyroidism occurred in 9.0% of men and 2.9% of women and anemia occurred in 3.8% of men and 8.8% of women. Finally, neutropenia and infections occurred in 2.6% of men and 5.9% of women, stroke of 6.4% of men and 1.5% of women, deregulation of diabetes II was presented 1.3% of men and 7.4% of women, and pneumonitis occurred in 3.8% of men and 2.9% of women.

As Table 4 shows, gender statistically significantly differentiated the most frequently reported treatment complications (χ2 = 30.38, df = 12, p = 0.002). Therefore, observed differences, particularly as presented in the previous Table 3, are statistically significant and gender has a significant impact on the treatment complications reported for patients.

Furthermore, it was examined whether gender differentiates the response to treatment, but also the rate of death of men and women. Gender was the independent variable and responding to treatment was the dependent variable. The results, presented in Tables 5 and 6, show that gender did not statistically significantly alter the response to treatment (p = 0.22).

Finally, it was examined whether gender as an independent variable differentiates patient deaths (Tables 7 and 8). Sex did not statistically affect the number of deaths (p = 0.12).

It was further examined whether the type of treatment of patients (chemotherapy or immunotherapy) statistically significantly altered the response to treatment, death rate, and treatment complications. The results, shown in Tables 9 to 14 below, showed that the type of treatment received had a statistically significant effect on treatment and treatment complications, but not the rate of death.

In particular, as shown in Table 9, 21.5% of those receiving chemotherapy and 10.6% of those receiving immunotherapy had stable disease. 4.7% of those receiving chemotherapy and 21.2% of those receiving immunotherapy had complete remission, and 32.9% of chemotherapy recipients and 45.5% of immunotherapy recipients had partial remission. Finally, 40.9% of those receiving chemotherapy and 22.7% of those receiving immunotherapy had progressively worsening disease.

Differences between patients receiving chemotherapy and those receiving immunotherapy were statistically significant (χ2 = 22.01, df = 3, p = 0.0005).

Tables 11 and 12 show the findings for the relationship of the treatment type to the percentage of deaths. The type of treatment received did not statistically affect the rate of death (p = 0.33, Table 12).

Finally, Tables 13 and 14 show the results for the effect of the treatment type on the complications of the treatment. In Table 13, lower limb neuropathy occurred in 22.6% of cases of chemotherapy and only 2.4% of cases of immunotherapy.

Pancytopaenia and infections occurred in 18.9% of cases of chemotherapy, and only 2.4% of cases of immunotherapy, respiratory failure II in 11.3% of chemotherapy cases and 7.3% of cases of immunotherapy, depression in 8.5% of cases of chemotherapy and only 2.4% of cases of immunotherapy, and hypothyroidism occurred in only 0.9% of cases of chemotherapy and 31.7% of cases of immunotherapy. The incidence of diabetes II occurred in 6.6% of chemotherapy cases and 7.3% of cases of immunotherapy, while gastrointestinal disturbances occurred in 10.4% of chemotherapy cases, but in none of the patients receiving immunotherapy. Hyperthyroidism, however, did not occur at all in the cases of chemotherapy but was reported in 22% of the cases of immunotherapy. Anemia occurred in 7.5% of cases of chemotherapy and 2.4% of cases of immunotherapy.

Neutropenia and infections occurred in 5.7% of chemotherapy cases, but in no immunotherapy patient, whereas stroke occurred in 4.7% of cases of chemotherapy and 2.4% of cases of immunotherapy. Diabetes II deregulation occurred in 2.8% of chemotherapy cases and 7.3% of cases of immunotherapy, and pneumonitis showed 12.2% of cases of immunotherapy but no chemotherapy patient (Table 13).

Differences observed between chemotherapy and immunotherapy patients analyzed in Table 13 were statistically significant (χ2 = 90, df = 12, p = 0.0005). Table 14 presents the statistically significant finding.

## Results

The conclusions that can be drawn for this study are the following. As for the medical data of the 218 patients in the study, 69% received chemotherapy and 31% were immunotherapy treated. The most common accompanying disease was chronic obstructive pulmonary disease (76%), coronary artery disease (30%), arterial hypertension (29%), and diabetes (29%) follow. Other concomitant diseases reported by several participating patients were mental illness (10%), chronic renal failure (9%), bronchial asthma (5%), hypothyroidism (5%) and cerebrovascular accident (3%). In total, 19% of the patients in this sample died.

Gender had a statistically significant effect on treatment complications, with the greatest differences observed in gastrointestinal disorders (occurring in only 16% of women), on the onset of diabetes II (men 10%, women 3%) and on deregulation diabetes mellitus (women 7%, men 1%), depression (women 10%, males 4%), hyperthyroidism (men 9%, females 3%) and anemia (women 9%, males 4%). Sex did not statistically affect the response to treatment or the number of deaths.

The type of treatment was statistically significant in responding to the treatment and complications of treatment, but not in the rate of death. Patients receiving chemotherapy and those receiving immunotherapy varied in all cases of treatment response, namely progressively worsening disease (41% chemotherapy, 23% immunotherapy), complete recession (21% immunotherapy, 5% chemotherapy) (225% chemotherapy, 11% immunotherapy) and partial remission (46% immunotherapy, 33% chemotherapy).

In addition, the type of treatment had a statistically significant effect on treatment complications, with the greatest differences being found in hypothyroidism (32% immunotherapy, 1% chemotherapy), lower limb neuropathy (23% chemotherapy, 2% immunotherapy), hyperthyroidism (10% chemotherapy, 0% immunotherapy), and finally pneumonitis (12% immunotherapy, 0% chemotherapy), in pancytopenia and infections (19% chemotherapy, 2% immunotherapy). The type of treatment received did not statistically affect the rate of death.

## Discussion

The conclusions that can be drawn for this study are the following. As for the medical data of the 218 patients in the study, 69% received chemotherapy and 31% were immunotherapy treated. The most common accompanying disease was chronic obstructive pulmonary disease (76%), coronary artery disease (30%), arterial hypertension (29%), and diabetes (29%) follow. Other concomitant diseases reported by several participating patients were mental illness (10%), chronic renal failure (9%), bronchial asthma (5%), hypothyroidism (5%) and cerebrovascular accident (3%). In total, 19% of the patients in this sample died.

Gender had a statistically significant effect on treatment complications, with the greatest differences observed in gastrointestinal disorders (occurring in only 16% of women), on the onset of diabetes II (men 10%, women 3%) and on deregulation diabetes mellitus (women 7%, men 1%), depression (women 10%, males 4%), hyperthyroidism (men 9%, females 3%) and anemia (women 9%, males 4%). Sex did not statistically affect the response to treatment or the number of deaths.

The type of treatment was statistically significant in responding to the treatment and complications of treatment, but not in the rate of death. Patients receiving chemotherapy and those receiving immunotherapy varied in all cases of treatment response, namely progressively worsening disease (41% chemotherapy, 23% immunotherapy), complete recession (21% immunotherapy, 5% chemotherapy) (225% chemotherapy, 11% immunotherapy) and partial remission (46% immunotherapy, 33% chemotherapy).

In addition, the type of treatment had a statistically significant effect on treatment complications, with the greatest differences being found in hypothyroidism (32% immunotherapy, 1% chemotherapy), lower limb neuropathy (23% chemotherapy, 2% immunotherapy), hyperthyroidism (10% chemotherapy, 0% immunotherapy), and finally pneumonitis (12% immunotherapy, 0% chemotherapy), in pancytopenia and infections (19% chemotherapy, 2% immunotherapy). The type of treatment received did not statistically affect the rate of death.

The results of the study showed that in the sample studied, ie from 119 men and 217 women, the gender of the patient significantly affected the adverse effects of the treatment. The percentage of patients undergoing immunotherapy was 30.7%, chemotherapy was administered to the remaining patients).

In both treatments, the side effects reported in the majority of cases were infections, leg lesion neuropathy, pancytopenia, respiratory failure II, depression, diabetes mellitus II, hypothyroidism, gastrointestinal disorders, hyperthyroidism, anemia, cerebrovascular accident and neutropenia.

Gender differences (occurring in only 16% of women), diabetes II (men 10%, women 3%) and deregulation of the sugar Diabetes II (women 7%, men 1%), depression (women 10%, men 4%), hyperthyroidism (men 9%, women 3%) and anemia (women 9%, males 4%). Sex did not statistically affect the response to treatment or the number of deaths.

Previous studies show that the adverse effects associated with immunotherapy do not appear immediately but occur after several days or even weeks of treatment, especially with PD-1 / PD-L1 inhibitors (4-10 weeks) 23. CTLA-4 inhibitors cause more serious side effects, which tend to occur earlier during treatment 23. Similarly, combined treatment with a CTLA-4 inhibitor and a PD-1 / PD-L1 inhibitor causes more serious side effects, occurring earlier 23.

Different immune responses between men and women and possible interaction with the hormonal system may affect how men and women benefit from immunotherapy or not. The literature shows that increased sensitivity of women to autoimmune disorders may also make them more likely to experience adverse effects associated with immunotherapy, possibly leading to a higher rate of discontinuation of therapy 24. The results of a recent study show that immune control inhibitors can improve the overall survival of both sexes with certain types of advanced cancers, such as melanoma and non-small cell lung cancer, but also that men have a greater therapeutic effect than in women 25. Despite the obvious biological and physiological difference between men and women and the extensive literature on the possible role sex plays in pharmacokinetics, pharmacodynamics, and the efficacy of the drug, new therapeutic approaches such as immunotherapy are rarely controlled by gender considerations.

Since control point inhibitors are associated with specific adverse events, efforts are under way to identify predictive biomarkers for the selection of patients who would have the greatest possible benefit from immunotherapy. Gender-related differences in the benefit of immunotherapies remain an issue that has not yet been investigated to the extent required to produce safe conclusions. Probably, the first study showing clearly significant heterogeneity in the efficacy of these inhibitors by gender of the patient is of Conforti et al. (2018) 25. In another study Botticelli et al., 2017 26, the exact same result is obtained. The relevance of the findings is enhanced by their consistency across all the subsets analyzed. The heterogeneity test for gender-related interaction, evaluated in each subgroup, was not significant and seems to support the findings of a previous survey. The increased effectiveness of immunotherapy in men versus female patients was evident in all types of cancer. Small cell lung cancer was the only type of cancer found to have a lack of gender differences. In such cases, for both male and female patients, ipilimumab appeared to be an ineffective therapeutic strategy in the treatment of small cell lung cancer.

Hoffner et al. (2018) reported that the patient's gender should be taken into account when assessing the balance between risk and benefit in the choice of treatment strategies, and that the design of future immunotherapy studies should guarantee increased inclusion of women in clinical tests 27.

From a recent systemic review and meta-analysis with 3803 patients who received immunotherapy with various agents (nivolumab 1534, pembrolizumab 1459, azetolizmumab 751) and a control group of 2873 patients who underwent chemotherapy with various agents (cetuximab 2476 and biological factor 397) . The study does not refer to the sex of the patients, however it is clarified that the overall study considered the gender of the patients. Immune system-related adverse reactions were reported in all studies. In particular, 214 patients reported hypothyroidism 214 (5.6%), 85 (2.2%) pneumonitis, 25 (0.7%) colitis, 6 (0.2%) hepatitis and 11 (0.3%) subfusitis.

From our study, and the literature, there are several common signs in the immunotherapy-induced side effects. However, although it is a study in the literature, the gender of the patients is a point of reference for the efficacy of the treatment and not for the differentiation in the induced side effects. Previous reports suggest that increased sensitivity of women to autoimmune disorders may also make them more likely to experience adverse effects associated with immunotherapy. Therefore, it is essential that the gender of the patient is taken into account when assessing the balance between the risk of adverse events and benefits in the selection of treatment strategies, and that future immunotherapy studies should be planned to guarantee increased inclusion of women in clinical trials to produce safer findings but also more effective treatments, with greater security/unwanted effects.

## Figures and Tables

**Table 1:** Patient comorbidities

	Frequency Display	Occurrence Rate
Chronic Obstructive Pulmonary Disease (COPD)	165	75.7
Coronary Disease	66	30.3
Hypertension	64	29.4
Diabetes	63	28.9
Mental Illness	21	9.6
Chronic Kidney Failure	20	9.2
Asthma	11	5.0
HypoThyroidism	11	5.0
Stroke	7	3.2
Congestive Heart Failure	5	2.3
Hyperlipidemia	5	2.3
Autoimmune Diseases	3	1.4
Gastroesophageal reflux	3	1.4
HyperThyroidism	3	1.4

**Table 2:** Treatment adverse effects

Treatment complication	Frequency	Percentage
Infections	29	14.2
Neuropathy of lower extremity	27	13.2
Pancytopenia	26	12.7
Respiratory Deficiency type II	20	9.8
Depression	17	8.3
Diabetes type II	16	7.8
HypoThyroidism	16	7.8
Gastrointestinal disorders	14	6.9
HyperThyroidism	13	6.4
Anemia	11	5.4
Stroke	9	4.4
Neutropenia	8	3.9
Skin rash / Dermatopathy	7	3.4
Decongestant diabetes	7	3.4
Pneumonitis	6	2.9
Colitis	5	2.5
Osteoporosis	4	2.0
Atrial Fibrillation	3	1.5
Ulcerative colitis	3	1.5
Chronic Kidney Failure type II	3	1.5
Gastroesophageal reflux	2	1.0
Thrombocytopenia	2	1.0
Heart failure	2	1.0
Mental Illness	2	1.0
Arthritis	1	0.5
Hypertension	1	0.5
Haemoperidium	1	0.5
Hemoptysis	1	0.5
Hemorrhagic rash	1	0.5
Intermittent claudication	1	0.5
Diverticulitis	1	0.5
Migrane	1	0.5
Hepatitis	1	0.5
Thrombophlebitis	1	0.5
Vertigo	1	0.5
COPD Exacerbation	1	0.5
Esophagitis	1	0.5
Bone Jaw Necrosis	1	0.5
Transient Stroke	1	0.5
Pulmonary Embolism	1	0.5
Chollageitis	1	0.5
**Total adverse effects**	**269**	**100.0**

**Table 3:** Most Common Adverse Effects per treatment based on sex

Rank order	Adverse effect	Male		Female		Total	
		Ν	%	Ν	%	Ν	%
1.	Neuropathy of lower extremity	14	17.9	10	14.7	24	16.4
2.	Pancytopenia - Infections	14	17.9	7	10.3	21	14.4
3.	Respiratory Distress Type II	9	11.5	6	8.8	15	10.3
4.	Depression	3	3.8	7	10.3	10	6.8
5.	HypoThyroidism	9	11.5	5	7.4	14	9.6
6.	Diabetes emergence	8	10.3	2	2.9	10	6.8
7.	Gastrointestinal disorders	0	0.0	11	16.2	11	7.5
8.	HyperThyroidism	7	9.0	2	2.9	9	6.2
9.	Anemia	3	3.8	6	8.8	9	6.2
10.	Neutropenia - Infections	2	2.6	4	5.9	6	4.1
11.	Stroke	5	6.4	1	1.5	6	4.1
12.	Deregulation of type II diabetes	1	1.3	5	7.4	6	4.1
13.	Pneumonitis	3	3.8	2	2.9	5	3.4
	**Total**	**78**	**100.0**	**68**	**100.0**	**146**	**100.0**

**Table 4:** Statistics χ^2^ between adverse effects treatment and sex

	Χ^2^	df	p
Pearson Chi-Square	30,378	12	0,002

**Table 5:** Response of treatment per sex

Treatment response	Male		Female		Total	
	Ν	%	Ν	%	Ν	%
Stable Disease	24	20.7	15	15.3	39	18.2
Complete Response	10	8.6	11	11.2	21	9.8
Partial Response	36	31.0	42	42.9	78	36.4
Progressive disease	46	39.7	30	30.6	76	35.5
**Total**	**116**	**100.0**	**98**	**100.0**	**214**	**100.0**

**Table 6:** Statistics χ^2^ differences between response and sex

	Χ^2^	df	p
Pearson Chi-Square	4.472	3	0.215

**Table 7:** Deaths per sex

	Male		Female		Total	
	Ν	%	Ν	%	Ν	%
Deaths	18	15.1	23	23.5	41	18.9
Alive	101	84.9	75	76.5	176	81.1
Total	119	100.0	98	100.0	217	100.0

**Table 8:** Statistics χ^2^ differences between death rate and sex

	χ^2^	df	p
Pearson Chi-Square	2.441	1	0.118

**Table 9:** Response per therapy

Treatment response	Chemotherapy		Immunotherapy		Total	
	Ν	%	Ν	%	Ν	%
Stable disease	32	21.5	7	10.6	39	18.1
Complete response	7	4.7	14	21.2	21	9.8
Partial response	49	32.9	30	45.5	79	36.7
Progresive disease	61	40.9	15	22.7	76	35.3
**Total**	**149**	**100.0**	**66**	**100.0**	**215**	**100.0**

**Table 10:** Statistics χ^2^ differences between response and treatment

	χ^2^	df	p
Pearson Chi-Square	22.009	3	0.0005

**Table 11:** Death rate per therapy

	Chemotherapy		Immunotherapy		Total	
	Ν	%	Ν	%	Ν	%
Deaths	31	20.5	10	14.9	41	18.8
Alive	120	79.5	57	85.1	177	81.2
**Total**	**151**	**100.0**	**67**	**100.0**	**218**	**100.0**

**Table 12:** Statistics χ^2^ differences between death rate and treatment

	χ^2^	df	p
Pearson Chi-Square	0.955	1	0.329

**Table 13:** Treatment adverse effects per therapy

Rank order	Adverse effect	Chemotherapy		Immunotherapy		Total	
		Ν	%	Ν	%	Ν	%
1.	Lower extremity neuropathy	24	22.6	1	2.4	25	17.0
2.	Pancytopenia - Infections	20	18.9	1	2.4	21	14.3
3.	Respiratory distress type II	12	11.3	3	7.3	15	10.2
4.	Depression	9	8.5	1	2.4	10	6.8
5.	Hypothyroidism	1	0.9	13	31.7	14	9.5
6.	Emergence of type II diabetes	7	6.6	3	7.3	10	6.8
7.	Gastrointestinal disorders	11	10.4	0	0.0	11	7.5
8.	HyperThyroidism	0	0.0	9	22.0	9	6.1
9.	Anemia	8	7.5	1	2.4	9	6.1
10.	Neutropenia - Infections	6	5.7	0	0.0	6	4.1
11.	Stroke	5	4.7	1	2.4	6	4.1
12.	Disregulation of type II diabetes	3	2.8	3	7.3	6	4.1
13.	Pneumonitis	0	0.0	5	12.2	5	3.4
	Total	106	100.0	41	100.0	147	100.0

**Table 14:** Statistics χ^2^ differences between adverse effects and treatment

	χ^2^	df	p
Pearson Chi-Square	90.003	12	0.0005
